# Electrophysiological Properties and Mechanical Sensitivity of Trigeminal Ganglionic Neurons That Innervate the Maxillary Sinus in Mice

**DOI:** 10.3390/ijms27062565

**Published:** 2026-03-11

**Authors:** Saurav Gupta, Amit Raj Sharma, Jennifer Ling, Frederick Godley, Jianguo Gu

**Affiliations:** 1 Department of Anesthesiology and Perioperative Medicine, School of Medicine, University of Alabama at Birmingham, Birmingham, AL 35294, USA; gupta@uab.edu (S.G.); amitrajsharma@uabmc.edu (A.R.S.); jenniferling@uabmc.edu (J.L.); 2 University Otolaryngology, Providence, RI 02905, USA

**Keywords:** maxillary sinus, pain, migraine, Nav1.9, Nav1.8, Piezo2 channels, mechanoreceptors

## Abstract

The maxillary sinus is frequently implicated in facial pain syndromes arising from infection, neoplasia, dental procedures, and, importantly, migraine, which can mimic “sinus headache” and contribute to misdiagnosis and inappropriate antibiotic use. Despite the clinical burden of chronic maxillary sinus pain, the sensory neuron subtypes that convey nociceptive and mechanosensory signals from the sinus mucosa remain incompletely defined. In this study, trigeminal ganglion (TG) neurons innervating the maxillary sinus (maxillary sinus TG neurons) were retrogradely labeled with the fluorescent dye DiD in mice and characterized using ex vivo patch-clamp electrophysiology and single-cell RT-PCR. Maxillary sinus TG neurons were found to be predominantly small-diameter, C-afferent nociceptors with electrophysiologic features including high thresholds, repetitive firing, and broad action potentials. Notably, maxillary sinus TG neurons formed a distinct molecular and functional subgroup: they expressed Nav1.9, while showing minimal Nav1.8 expression and limited overlap with Nav1.8-positive nociceptor populations. A majority of maxillary sinus TG neurons were mechanically responsive, generating mechanically activated currents with heterogeneous adaptation profiles, and a subset expressed the mechanoreceptor Piezo2. Collectively, these findings identify maxillary sinus TG neurons as a specialized population of Nav1.9-enriched C-afferent nociceptors with mechanosensitive properties, providing a mechanistic framework for pressure-evoked sinus pain. This work advances the neurobiological basis of sinus-related pain and suggests that Nav1.9 and mechanoreceptor pathways may be potential therapeutic targets for conditions in which sinus symptoms overlap with migraine and other craniofacial pain disorders.

## 1. Introduction

The paranasal sinuses are a group of air-filled cavities within the craniofacial bones that drain into the nasal passages. Among these, the paired maxillary sinuses are the largest, occupying the space inferior to the orbits, lateral to the nasal cavity, and superior to the maxillary dentition. These structures are thought to reduce skull weight, enhance vocal resonance, and provide mechanical protection to adjacent facial structures. The sinus mucosa produces mucus that humidifies inspired air and contributes to immune defense within the upper airway [1]. Each maxillary sinus drains through the long and narrow hiatus semilunaris, located in the superior aspect of the sinus and opening into the middle meatus. This anatomically constrained drainage pathway renders the maxillary sinuses particularly susceptible to obstruction, leading to impaired mucus clearance, accumulation of secretions, and secondary infection [1,2].

Under pathological conditions, the maxillary sinuses can become a source of noxious pressure and pain [3]. Inflammatory processes are the most common cause of maxillary sinus pathology, typically triggered by viral or bacterial infections, although fungal infection, allergic rhinitis, nasal polyposis, and trauma may also contribute [4,5,6]. When patients report a “sinus headache,” they often describe dull facial pressure or pain around the eyes, cheeks, or forehead, sometimes accompanied by nasal symptoms. However, most of the time, the sinus headache is not due to a sinus infection. While persistent infection or inflammation may result in chronic pain and pressure syndromes [5,7], several studies show that 80–90% of people who self-diagnose or are clinically diagnosed with a sinus headache meet criteria for migraine or probable migraine and respond to migraine therapies [8,9,10,11]. Furthermore, most studies indicate a weak correlation between sinus CT findings and symptoms. In a 2022 study of 1849 patients with no sinus symptoms [12], one in three patients (33%) had an abnormal finding on sinus CT scan. Most of the affected sinuses were the maxillary. In another study of 53 patients with symptoms meeting the diagnostic criteria for CRS scans (predominantly posterior rhinorrhea and headache/facial pain) and negative (normal) CT (*p* < 0.001), 70% met the ICHD-3 criteria for migraine [13]. Migraines can produce nasal congestion and posterior rhinorrhea through activation of the superior salivatory nucleus and parasympathetic outflow to the turbinate vasculature and nasal secretory cells [14]. A major challenge in the management of sinus pain and pressure is diagnostic uncertainty. Misdiagnosis frequently results in delays in appropriate treatment and the use of ineffective or unnecessary interventions, including antibiotics or surgical procedures [15,16,17].

Understanding the sensory innervation of the paranasal sinus mucosa is crucial for explaining why patients may experience facial pain or pressure despite minimal objective evidence of sinus disease. Yet this knowledge remains incomplete. The maxillary sinuses are primarily innervated by the maxillary division (V2) of the trigeminal nerve, which conveys sensory information from this region. Based on conduction velocity, trigeminal afferent fibers can be classified as C-, Aδ-, or Aβ-afferent fibers. Functionally, C- and Aδ-afferent fibers primarily mediate nociception, whereas Aβ-afferent fibers are predominantly associated with low-threshold mechanosensation [18,19]. Nav1.8 and Nav1.9 are tetrodotoxin-resistant (TTX-R) voltage-gated sodium channels that are preferentially expressed in nociceptive C- and Aδ-afferent fiber populations. Nav1.8 channels play a critical role in the generation and maintenance of nociceptive signaling, particularly under inflammatory or injurious conditions [20]. Nav1.9 channels are selectively expressed in distinct subsets of nociceptive afferents and are important regulators of somatosensory neuron excitability [21]. Currently, the specific classes of trigeminal afferent fibers innervating the maxillary sinuses, as well as their electrophysiological properties, remain poorly defined. The maxillary sinuses can detect and respond to mechanical stimuli, including pressure changes, touch, and tissue deformation [22,23]. Mechanical pain and pressure are hallmark symptoms of sinus pathology, such as sinusitis. Piezo2 channels are well-established mechanoreceptors involved in touch sensation [24,25] and have been implicated in mechanical allodynia under inflammatory conditions [26]. However, whether Piezo2 channels also function as mechanotransducers in trigeminal afferent fibers innervating the maxillary sinuses remains unclear.

## 2. Results

**Figure 1:**
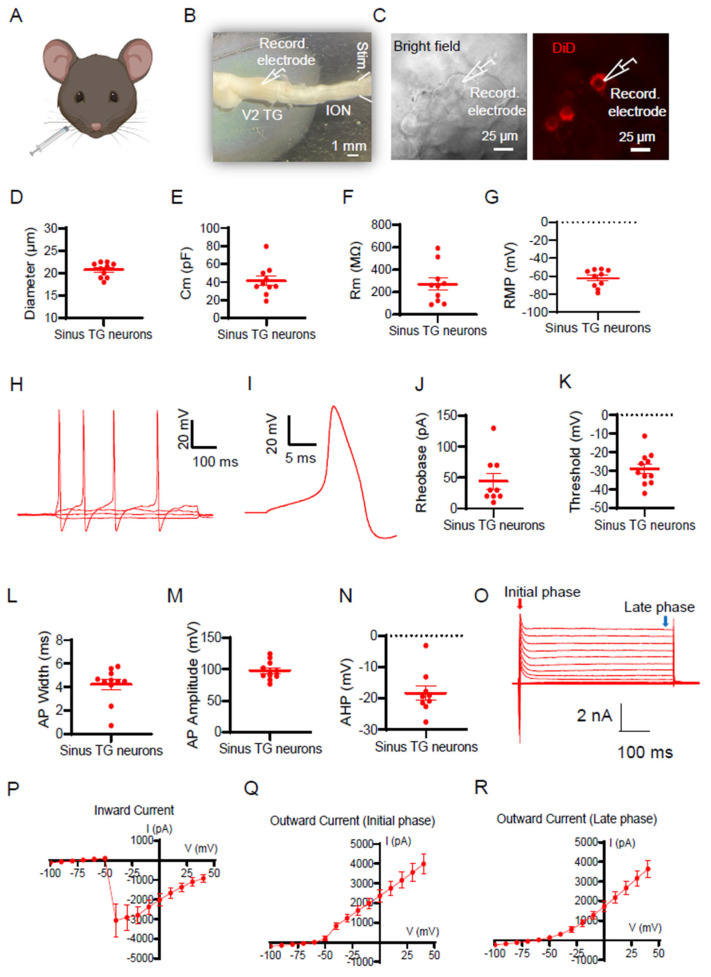
Electrophysiological properties of TG neurons that innervate the maxillary sinus of mice. (**A**) Illustration of retrograde labeling of TG neurons that innervate the maxillary sinus (maxillary sinus TG neurons). DiD was microinjected into the maxillary sinus of mice. (**B**) Ex vivo TG preparation made from DiD-injected mice and the setting for patch-clamp recordings. (**C**) DiD-labeled TG neurons in the ex vivo TG preparation were observed under the bright-field (left panel) and fluorescent-field (right panel). (**D**–**G**) Passive membrane properties of sinus TG neurons, including soma diameter (**D**), membrane capacitance (Cm, **E**), membrane input resistance (Rm, **F**), and resting membrane potential (RMP, **G**). (**H**) Representative trace showing multiple action potentials evoked by a 500-ms depolarizing current step in a sinus TG neuron. (**I**) Representative trace of an AP from a sinus TG neuron. APs were evoked by the injections of depolarizing currents via patch-clamp recording electrodes. (**J**–**N**) Active membrane properties of sinus TG neurons, including AP rheobase (**J**), AP threshold (**K**), AP width (**L**), AP amplitude (**M**), and afterhyperpolarization (AHP) amplitude (**N**). (**O**) Representative races of voltage-activated currents recorded from sinus TG neurons. (**P**) Current–voltage (I-V) relationship of voltage-activated inward Na^+^ currents in sinus TG neurons. (Q&R) I-V relationships of the early phase (**Q**) and late phase (**R**) outward K^+^ currents in sinus TG neurons. Data represent individual observations with mean ± SEM.

To identify TG neurons innervating the maxillary sinus for electrophysiological recordings, we first injected the fluorescent dye DiD into the maxillary sinus of C57BL/6J mice to retrograde-label these TG neurons (Figure 1A). These labeled TG neurons are referred to as sinus TG neurons in the present study. Ex vivo TG preparations were then made (Figure 1B), and whole-cell patch-clamp recordings were performed on sinus TG neurons (Figure 1C). Sinus TG neurons had small sizes, at 20.8 ± 0.5 μm (n = 10; Figure 1D) in diameter. Their membrane capacitance (Cm) was 41.3 ± 5.3 pF (n = 10; Figure 1E), membrane input resistance (Rm) was 269.6 ± 53.9 MΩ (n = 10; Figure 1F), and resting membrane potential (RMP) was −62.3 ± 3.1 mV (n = 10; Figure 1G). To assess their active membrane properties, we applied depolarizing current steps in current-clamp mode to the sinus TG neurons. Most sinus TG neurons fired multiple action potentials in response to a 500-ms depolarizing current injection (Figure 1H). AP displayed a shoulder in the repolarization phase (Figure 1I). These TG neurons showed small AP rheobase of 44.4 ± 12.9 pA (n = 9) (Figure 1J), high AP threshold (less negative) of −29.0 ± 2.6 mV (Figure 1K), broad AP width of 4.2 ± 0.4 ms (Figure 1L), high AP amplitude of 98.1 ± 4.5 mV (Figure 1M), and large afterhyperpolarization (AHP) amplitude of −18.3 ± 2.3 mV (Figure 1N). To investigate voltage-gated ion channel activity, we performed whole-cell voltage-clamp recordings on sinus TG neurons. Depolarizing voltage steps evoked fast inward Na^+^ currents followed by outward K^+^ currents (Figure 1O). The current–voltage (I–V) relationship for inward Na^+^ currents is shown in Figure 1P, and outward K^+^ currents were analyzed at both early (Figure 1Q) and late (Figure 1R) phases of the voltage step.

**Figure 2:**
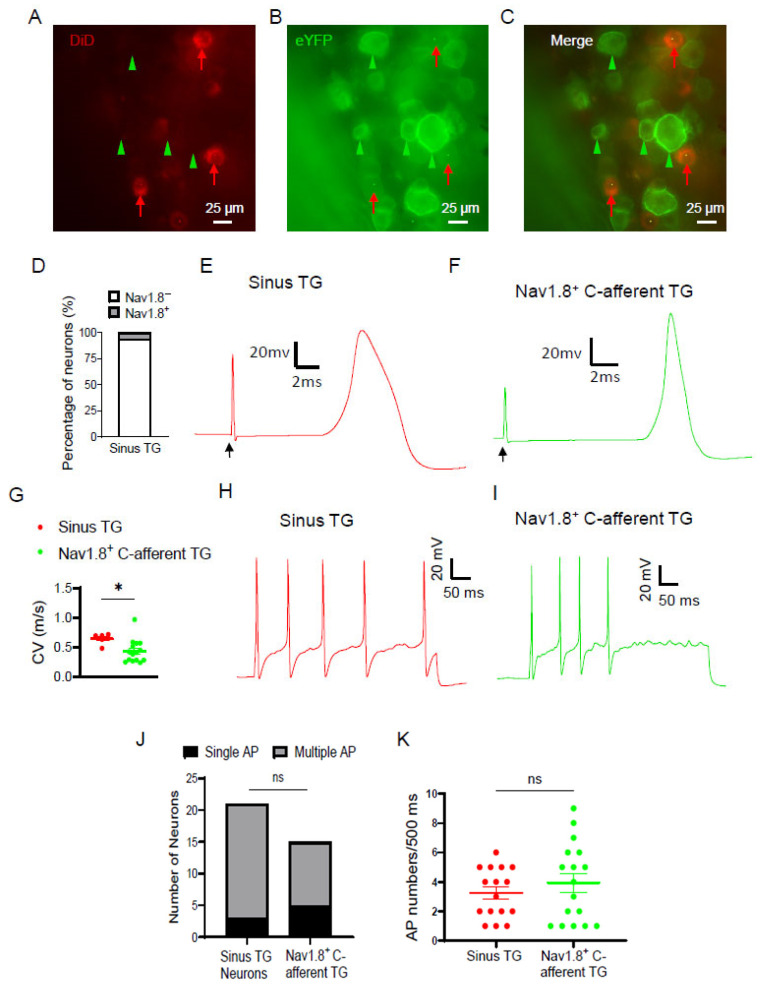
Comparison of properties between sinus TG neurons and Nav1.8-positive C-afferent TG neurons. DiD was injected into the maxillary sinus of Nav1.8-ChR2/eYFP mice, and 5 days later, TGs were harvested and ex vivo TG preparations were made. (**A**) Image shows sinus TG neurons labeled with DiD (red arrows indicated). (**B**) Image shows eYFP-positive, i.e., Nav1.8-positive (Nav1.8^+^) TG neurons. Four of them are indicated by green arrowheads. B is the same field as A. (**C**) A merged image of A and B shows that DiD-labeled TG neurons (sinus TG neurons) were not Nav1.8^+^ TG neurons. (**D**) Percentage of sinus TG neurons that were Nav1.8-positive and Nav1.8-negative. (**E**,**F**) Representative traces of APs recorded from a sinus TG neuron (**E**) and a Nav1.8^+^ C-afferent TG neuron (**F**). The APs were evoked by electrical stimulation applied to the infraorbital (V2) nerves (see Figure 1B). Arrows indicate stimulation artifacts. (**G**) Conduction velocity (CV) of sinus TG neurons (red symbols) and Nav1.8^+^ C-afferent TG neurons (green symbols). (**H**,**I**) Representative traces of repetitive AP firing in a sinus TG neuron (**H**) and a Nav1.8^+^ C-afferent TG neuron (**I**). APs were elicited by the injection of depolarizing currents at 2× rheobase for a duration of 500 ms. (**J**) Number of neurons displaying multiple (grey) or single (black) AP firing for sinus TG neurons (left) and Nav1.8-positive C-afferent TG neurons (right). (**K**) AP numbers evoked by 2× rheobase of depolarizing currents for 500 ms in sinus TG neurons and Nav1.8-positive C-afferent TG neurons. Data are presented as individual observations with mean ± SEM, ns, not significant, * *p* < 0.05, unpaired Student’s *t*-test, or Chi-square test.

We next used Nav1.8-cre-eYFP mice to investigate whether sinus TG neurons were Nav1.8-positive (Nav1.8^+^) TG neurons, since Nav1.8^+^ TG neurons were found to be mainly nociceptors. In this set of Experiments, sinus TG neurons were retrograde-labeled by injecting DiD into the maxillary sinus of the Nav1.8-cre-eYFP mice, and ex vivo TG preparations were made 5 days after the DiD injection. Surprisingly, we found that almost all sinus TG neurons that showed red fluorescence were negative for eYFP (Figure 2A–D). This indicated that sinus TG neurons were mostly not Nav1.8^+^ TG neurons. Of 83 DiD-positive neurons, 78 neurons (94%) were negative for eYFP, while only 5 neurons (6%) co-expressed eYFP (Figure 2D). These data indicate that sinus TG neurons represent a subpopulation of nociceptors distinct from Nav1.8-positive afferent nociceptors. We then investigated the electrophysiological properties of sinus TG neurons and compared them with those of Nav1.8-positive C-afferent TG neurons. In this set of experiments, whole-cell patch-clamp recordings were performed on DiD-positive sinus TG neurons and eYFP-positive/DiD-negative small-diameter TG neurons located in the V2 region in the ex vivo TG preparations. Conduction velocity (CV) was determined by stimulating the infraorbital (V2) nerve while APs were recorded in the soma of the selected TG neurons (Figure 2E,F). Both sinus TG neurons and small-sized Nav1.8-positive afferent neurons exhibited CVs < 1.0 m/s, indicative of C-afferent TG neurons (Figure 2E–G). Overall, sinus TG neurons displayed a significantly higher CV (0.64 ± 0.04 m/s, n = 6) compared to Nav1.8^+^ C-afferent TG neurons (0.43 ± 0.05 m/s, n = 15; Figure 2G). When prolonged depolarizing step currents were injected in the somas of these neurons, most sinus TG neurons and Nav1.8^+^ C-afferent TG neurons fired multiple APs (Figure 2H–J). The proportion of neurons exhibiting repetitive firing did not differ significantly between the two groups (Figure 2J). The number of action potentials evoked at twice the rheobase over 500 ms was comparable: 3.25 ± 0.42 (n = 16) for sinus TG neurons and 3.94 ± 0.65 (n = 17) for Nav1.8^+^ C-afferent TG neurons (Figure 2K).

**Figure 3:**
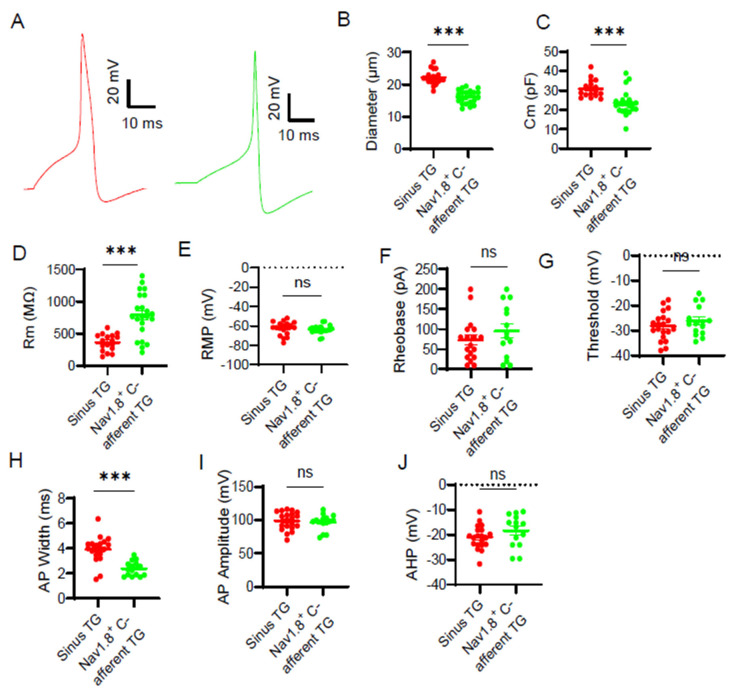
Comparison of passive and active membrane properties between sinus TG neurons and Nav1.8-positive C-afferent TG neurons (**A**) Representative traces of APs from a sinus TG neuron (left) and a Nav1.8^+^ C-afferent TG neuron (right). APs were evoked by the injections of depolarizing currents via patch-clamp recording electrodes. (**B**–**E**) Passive membrane properties of recorded neurons, including soma diameter (**B**), membrane capacitance (Cm, **C**), input resistance (Rm, **D**), and resting membrane potential (RMP, **E**). (**F**–**J**) Active membrane properties, including AP rheobase (**F**), AP threshold (**G**), AP width (**H**), AP amplitude (**I**), and afterhyperpolarization (AHP) amplitude (**J**). Data are presented as individual observations and mean ± SEM, ns, not significant, *** *p* < 0.001, unpaired Student’s *t*-test.

We further compared the passive and active membrane properties between sinus TG neurons and Nav1.8^+^ TG neurons. Figure 3A shows representative action potentials (APs) recorded from a sinus TG neuron (Left) and a Nav1.8^+^ C-afferent TG neuron in response to depolarizing current injection in the somas. Sinus TG neurons exhibited significantly larger soma diameters (22.2 ± 0.5 μm, n = 19) compared to Nav1.8^+^ C-afferent TG neurons (16.2 ± 0.4 μm, n = 22; Figure 3B). Consistently, membrane capacitance (Cm) was also greater in sinus TG neurons (30.9 ± 1.1 pF, n = 17) than in Nav1.8^+^ C-afferent TG neurons (23.5 ± 1.4 pF, n = 22; Figure 3C). Membrane input resistance (Rm) was significantly lower in sinus TG neurons (365.0 ± 31.8 MΩ, n = 17) than in Nav1.8^+^ C-afferent TG neurons (786.9 ± 72.4 MΩ, n = 22, Figure 3D). Resting membrane potential (RMP) was similar between the two groups, with sinus TG neurons at −62.5 ± 1.6 mV (n = 19) and Nav1.8^+^ C-type neurons at −63.7 ± 0.9 mV (n = 22; Figure 3E). AP rheobase was not significantly different between the two groups, with sinus TG neurons showing 72.8 ± 12.4 pA (n = 18), Nav1.8^+^ C-type neurons at 95.4 ± 17.7 pA (n = 14; Figure 3F). AP threshold was −28.1 ± 1.3 mV in sinus TG neurons and −26.0 ± 1.5 mV in Nav1.8-positive C-afferent TG neurons, and was not significantly different between them (Figure 3G). Notably, AP width was significantly greater in sinus TG neurons (3.92 ± 0.23 ms, n = 20) compared to Nav1.8^+^ C-afferent TG neurons (2.38 ± 0.15 ms, n = 15; *p* < 0.001; Figure 3H). AP amplitude was similar between the two groups (98.7 ± 3.0 mV in sinus TG neurons vs. 97.4 ± 3.5 mV in Nav1.8^+^ C-afferent TG neurons; Figure 3I). Afterhyperpolarization (AHP) amplitude was not significantly different between the sinus TG neurons (−21.0 ± 1.0 mV, n = 20) and the Nav1.8^+^ C-afferent neurons (−18.4 ± 1.7 mV, n = 14; Figure 3J). These results indicate that while sinus TG neurons and Nav1.8^+^ C-afferent neurons share some electrophysiological features, they differ significantly in soma size, membrane capacitance, input resistance, and AP width.

**Figure 4:**
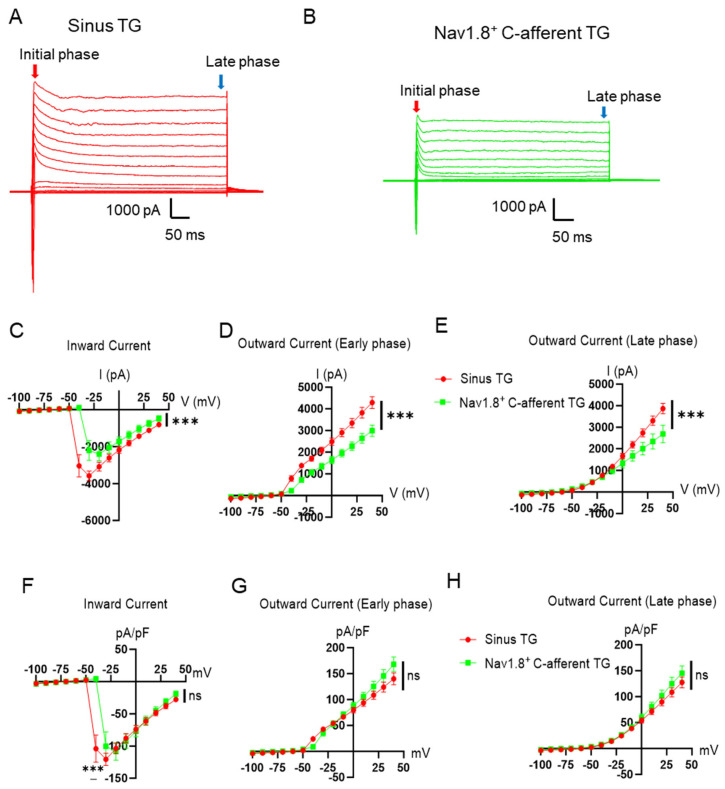
Voltage-activated inward Na^+^ and outward K^+^ currents in sinus TG neurons and Nav1.8-positive C-afferent TG neurons. (**A**,**B**) Representative races of voltage-activated currents recorded from sinus TG neurons (**A**) and Nav1.8^+^ C-afferent TG neurons (**B**). (**C**) Current–voltage (I-V) relationship of voltage-activated inward Na^+^ currents in maxillary sinus TG neurons (red; n = 10) and Nav1.8^+^ C-afferent TG neurons (green; n = 9). (**D**,**E**) I-V relationships of the early phase (**D**) and late phase (**E**) outward K^+^ currents in maxillary sinus TG neurons (red; n = 10) and Nav1.8^+^ C-afferent TG neurons (green; n = 9). (**F**) Similar to C, except the inward Na^+^ currents were normalized to the membrane capacitance. (**G**,**H**) Similar to D and E, except the outward K^+^ currents were normalized to the membrane capacitance. Data are presented as individual observations with mean ± SEM; ns, not significant; *** *p* < 0.001; two-way ANOVA with Šidák’s post hoc test.

We next investigated voltage-activated Na^+^ and K^+^ currents in sinus TG neurons and Nav1.8^+^ C-afferent TG neurons using whole-cell voltage-clamp recordings. Depolarizing voltage steps elicited robust, fast inward Na^+^ currents, followed by outward K^+^ currents, in both neuronal populations (Figure 4A,B). Current–voltage (I-V) curve revealed that sinus TG neurons exhibited significantly larger inward Na^+^ currents compared to Nav1.8^+^ C-afferent TG neurons (Figure 4C). Similarly, both early-phase and late-phase outward K^+^ currents were significantly higher in sinus TG neurons than in Nav1.8^+^ C-afferent TG neurons (Figure 4D,E). Since cell sizes were different between sinus TG neurons and Nav1.8^+^ C-afferent TG neurons, we normalized the voltage-activated inward and outward currents with their membrane capacitance. After the normalization, maxillary sinus TG neurons still exhibited significantly larger inward Na^+^ currents than Nav1.8^+^ C-afferent TG neurons at −40 mV voltage step (Figure 4F). For the early-phase and late-phase outward K^+^ currents, there were no significant differences between the two groups after the normalization (Figure 4G,H).

**Figure 5:**
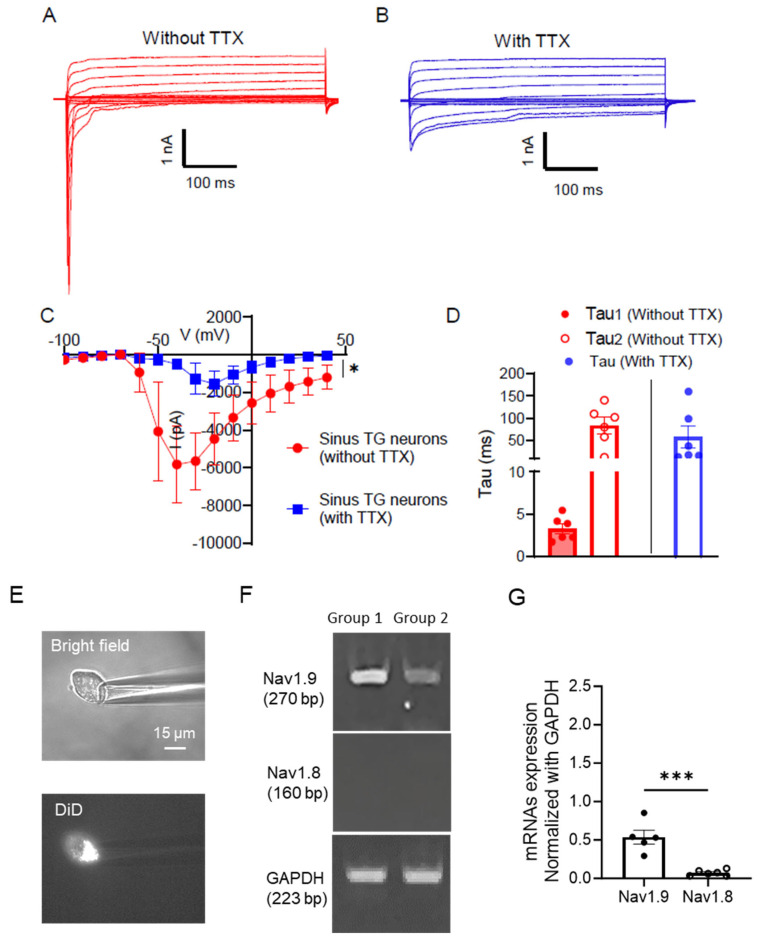
TTX-resistant voltage-activated Na^+^ currents and expression of Nav1.9 in sinus TG neurons (**A**) Representative traces of voltage-activated inward Na^+^ currents recorded from a sinus TG neuron. Recordings were performed in the absence of tetrodotoxin (TTX). Voltage-gated K^+^ channel blockers Cs^+^ (135 mM in recording electrode internal solution), 10 mM TEA, and 1 mM 4-AP were present in the bath solution. (**B**) Representative traces of the tetrodotoxin-resistant (TTX-R) voltage-activated Na^+^ currents recorded from the same sinus TG neuron in A. Recording conditions were similar to A, except in the presence of 500 nM TTX. (**C**) Current–voltage (I-V) relationship of the voltage-activated Na^+^ currents in sinus TG neurons (n = 6) in the absence (red) and presence (blue) of 500 nM TTX. (**D**) Inactivation kinetics showing two-exponential decay (Tau_1_ and Tau_2_) in total Na^+^ currents (in the absence of TTX) and single exponential decay (Tau) in TTX-R Na^+^ currents (in the presence of TTX). The voltage was at −20 mV for evoking inward currents to measure tau. (**E**) Top, bright-field image shows pipette aspiration of a single DiD-labeled sinus TG neuron. Bottom, corresponding DiD fluorescence image of the single sinus TG neuron. (**F**) Sample gel images show expression of Nav1.9 mRNAs and Nav1.8 mRNAs in sinus TG neurons in two groups of experiments. (**G**) Quantification of Nav1.9 and Nav1.8 mRNA levels normalized to GAPDH mRNAs in sinus TG neurons. Data are presented as individual observations with mean ± SEM, ns, not significant, * *p* < 0.05, *** *p* < 0.001, unpaired Student’s *t*-test or two-way ANOVA with Šidák’s post-hoc test.

Given that sinus TG neurons were primarily Nav1.8-negative C-afferent TG neurons distinct from the Nav1.8^+^ C-afferent TG neurons, we examined whether sinus TG neurons expressed tetrodotoxin-resistant (TTX-R) Na^+^ channels. Whole-cell voltage-clamp recordings were performed on sinus TG neurons. Voltage-gated K^+^ channels were pharmacologically blocked using intracellular cesium (135 mM Cs^+^), and extracellular tetraethylammonium (TEA, 10 mM) and 4-aminopyridine (4-AP, 1 mM). The experiments were performed in the absence and presence of 500 nM tetrodotoxin (TTX). In the absence of TTX, depolarizing voltage steps evoked large inward Na^+^ currents in sinus TG neurons (Figure 5A–C). Upon application of 500 nM TTX, the fast inward Na^+^ current component was abolished, but slow inward Na^+^ currents remained (Figure 5B,C), indicating the presence of TTX-R Na^+^ channels in sinus TG neurons. Analysis of Na^+^ current inactivation kinetics revealed a two-exponential decay in the absence of TTX, with the decay time constant for the fast component (Tau_1_) of 3.3 ± 0.6 ms and the slow component (Tau_2_) of 83.5 ± 18.3 ms. In the presence of TTX, Na^+^ current inactivation displayed only a slow decay component fitting into a single exponential decay with a time constant (Tau) of 56.9 ± 24.5 ms (Figure 5D). The slow decay and TTX-R property of the inward Na^+^ currents are consistent with the kinetics of Nav1.9 channels. To further support this idea, we performed single-cell RT-PCR on sinus TG neurons. Following acute dissociation, DiD-labeled neurons were individually aspirated into micropipettes and collected in test tubes (Figure 5E). RT-PCR results revealed Nav1.9 expression, with minimal or no Nav1.8 expression (Figure 5F). Quantitative analysis showed that Nav1.9 mRNA levels, normalized to GAPDH, were significantly higher (0.54 ± 0.09, n = 5) than Nav1.8 mRNA levels (0.07 ± 0.02, n = 6; *p* < 0.001; Figure 5G). These results indicate that sinus TG neurons express Nav1.9 channels that account for TTX-R Na^+^ currents.

**Figure 6:**
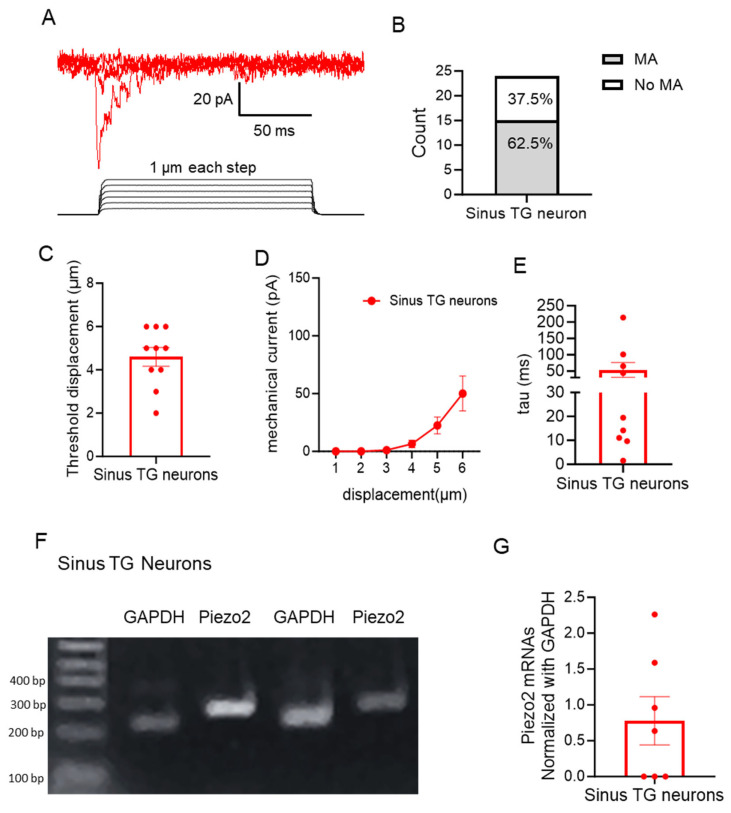
Mechanically activated currents and Piezo2 expression in sinus TG neurons. (**A**) Representative traces of mechanically activated (MA) currents recorded from a sinus TG neuron in response to stepwise membrane displacements. The MA currents were elicited by the graded membrane displacements with steps of 1 μm increments. (**B**) Proportion of sinus TG neurons responding to the mechanical stimulation with MA currents. (**C**) Displacement thresholds for evoking MA currents in sinus TG neurons. (**D**) Summary data of the MA current amplitudes recorded from sinus TG neurons in response to increased membrane displacement (n = 9). The MA currents were elicited by the graded membrane displacements from 1 to 6 μm. (**E**) Decay time constants (Tau) of MA currents in sinus TG neurons. (**F**) Sample gel images show mRNA expression of Piezo2 and GAPDH in sinus TG neurons of two sets of experiments. (**G**) Quantification of Piezo2 mRNA expression normalized to GAPDH mRNA in sinus TG neurons. Data are presented as individual observations with mean ± SEM.

We determined whether sinus TG neurons are mechanically sensitive. In this set of experiments, whole-cell voltage-clamp recordings were performed in sinus TG neurons. Stepwise membrane displacements were applied to the sinus TG neurons to evoke mechanically activated (MA) currents (Figure 6A). Among the recorded sinus TG neurons, 15 out of 24 (62.5%) responded to mechanical stimulation with MA currents, while 9 out of 24 (37.5%) did not show detectable MA currents with displacement up to 8 µm (Figure 6B). Membrane displacement over 8 µm usually disrupted the tight seal between the recording electrode and the recorded TG neurons. The mechanical threshold, defined as the minimal membrane displacement required to elicit MA currents, was 4.6 ± 0.4 µm (n = 10; Figure 6C). Figure 6D shows the relationship between MA currents and membrane displacements. Analysis of the decay kinetics of MA currents revealed three adaptation profiles, classified by the decay time constant (Tau): rapidly adapting (Tau ≤ 10 ms), intermediately adapting (10 ms < Tau ≥ 30 ms), and slowly adapting (Tau > 30 ms) [27]. Among the nine sinus TG neurons that responded to mechanical stimulation, two exhibited rapid adaptation, three showed intermediate adaptation, and four were slowly adapting (Figure 6E). To explore whether Piezo2 channels may mediate MA currents in sinus TG neurons, we performed single-cell RT-PCR to assess Piezo2 expression in sinus TG neurons. In this set of experiments, after acute dissociation, DiD-labeled sinus TG neurons were individually collected (6 neurons per group) for RT-PCR (see Figure 5E). Piezo2 mRNAs, normalized to GAPDH, were detected in four out of seven pooled groups, while three groups showed no detectable Piezo2 mRNAs (Figure 6G).

## 3. Discussion

Elucidating the electrophysiological and mechanotransducive properties of trigeminal afferent fibers innervating the maxillary sinuses is essential for advancing our understanding of sinus-related pain across pathological states. In the present study, using retrograde tracing to label TG neurons innervating the maxillary sinuses (maxillary sinus TG neurons), we combined patch-clamp electrophysiology with molecular profiling to characterize their functional and phenotypic properties. We demonstrate that sinus TG neurons constitute a distinct subpopulation of C-afferent nociceptors. These TG neurons are characterized by a predominance of Nav1.9 expression and minimal or absent Nav1.8 expression. A substantial proportion of these TG neurons respond to mechanical stimulation with MA currents, and Piezo2 mRNA is expressed in a subset of this population. Collectively, these findings identify sinus TG neurons as a previously unrecognized subtype of C-afferent nociceptors.

Sinus TG neurons exhibit hallmark electrophysiological features of C-afferent nociceptors, including high action potential (AP) thresholds, large AP amplitudes, and broad AP waveforms with a characteristic shoulder during depolarization. Additionally, most sinus TG neurons fire repetitively in response to sustained depolarizing current injection, a property commonly observed in C-afferent nociceptors [28,29]. While classical C-afferent nociceptors typically express Nav1.8 [30], sinus TG neurons were predominantly Nav1.8-negative. Notably, the afferents of sinus TG neurons exhibited conduction velocities slightly but significantly faster than those of Nav1.8^+^ C-afferent TG neurons. Broad APs of sinus TG neurons, particularly those with a depolarization shoulder, are a well-recognized electrophysiological signature of unmyelinated C-afferent nociceptors [29,31,32,33], reflecting complex interactions among ion channels during repolarization, including contributions from voltage-gated Ca^2+^ channels [34]. Voltage-clamp recordings revealed that sinus TG neurons exhibit robust inward Na^+^ currents and outward K^+^ currents that exceed those observed in Nav1.8^+^ C-afferent TG neurons. After normalization to membrane capacitance, Na^+^ current density was slightly higher in sinus TG neurons, whereas outward K^+^ current density did not differ significantly between the two populations. Because voltage-gated K^+^ channels are key determinants of AP repolarization and waveform duration [35], the lack of differences in K^+^ current density suggests that additional mechanisms, e.g., voltage-gated Ca^2+^ currents, may contribute to the broader APs observed in sinus TG neurons.

Somatosensory neurons express multiple isoforms of voltage-gated Na^+^ channels that differ in gating properties and tetrodotoxin (TTX) sensitivity [36]. Nociceptors typically co-express TTX-sensitive and TTX-resistant Na^+^ channels, with Nav1.8 and Nav1.9 representing the major TTX-resistant isoforms [36]. In contrast to classical C-afferent nociceptors, sinus TG neurons were largely Nav1.8-negative. Voltage-clamp recordings revealed the presence of TTX-resistant Na^+^ currents with slow inactivation kinetics, consistent with Nav1.9 channel expression in sinus TG neurons. This electrophysiological profile was corroborated by molecular analyses demonstrating high Nav1.9 expression and minimal Nav1.8 expression in sinus TG neurons. These findings reveal a molecular distinction between sinus TG neurons and classical Nav1.8^+^ C-afferent nociceptors. Notably, we observed repetitive firing patterns in these neurons despite their minimal Nav1.8 expression. The high expression of Nav1.9 may be the cause to enhance neuronal excitability and repetitive firing in maxillary sinus TG neurons.

Nav1.9 has been implicated in trigeminal neuropathic pain, contributing to orofacial hypersensitivity independent of Nav1.8-mediated mechanisms [21]. Emerging evidence also suggests a role for Nav1.9 in migraine pathophysiology, potentially through enhanced trigeminal excitability and pain amplification [37]. In the nasal mucosa, C-afferent fibers are known to innervate the subepithelial, glandular, and vascular regions [38]. C-afferent fibers are described as wrapping around small blood vessels and, through their mechanoreceptors, respond to swelling of blood vessels either through allergic release of neurotrophins, neuropeptides, vascularly delivered CGRP during a migraine attack, barometric pressure changes, or autonomic activation [39]. In this context, the selective expression of Nav1.9 in sinus TG neurons raises the possibility that several conditions can lead to the stretching or distortion of these C-afferent nociceptors through vascular engorgement, mucosal edema, or neurogenic inflammation and the generation of the sensation of dull facial pain or pressure.

A notable finding of this study is that a substantial proportion of sinus TG neurons responded to mechanical stimulation, exhibiting MA currents with diverse adaptation kinetics. This heterogeneity suggests the involvement of multiple mechanotransduction mechanisms. Rapidly adapting MA currents are believed to be mediated by Piezo2 channels, which are essential for normal touch sensation [24,27]. We identified Piezo2 expression in a subset of sinus TG neurons, supporting its contribution to mechanotransduction in these cells. Beyond its role in innocuous mechanosensation, Piezo2 has also been implicated in mechanical allodynia under inflammatory conditions [26,40]. Accordingly, Piezo2-mediated MA currents in sinus TG neurons may contribute to both physiological sensing of sinus pressure and pathological mechanical pain. The molecular identity of the channels underlying the slowly adapting MA currents observed in other sinus TG neurons remains to be determined and warrants further investigation.

A comparison of fiber types, nociceptors, sodium ion channels, mechanosensitive ion channels, and TRP channels (Table 1) shows how the fundamental components of the sensory system have developed to meet the needs of specific anatomic locations and create different symptoms, such as sharp dental pain, touch-sensitive pain of the cornea, temperature or chemical sensitivity in the nose or throbbing pain of the meninges. Even two distinct regions innervated by trigeminal nerves, the dura and nasal mucosa, use similar molecular tools, but in different concentrations, contexts, and functional roles. Both rely on C-afferent fiber-driven nociception, Nav1.9, and silent nociceptors, yet elicit different sensations in patients. Future studies should examine the central projections, synaptic targets, and behavioral relevance of sinus-innervating C-afferent nociceptors, as well as their roles in convergence and central sensitization in the generation of facial pain and pressure. Such work may clarify the range of mechanisms of peripheral sensitization, reveal novel mechanisms underlying sinus-associated pain disorders, including migraine, and identify new therapeutic targets, such as Nav1.9 blockers, Piezo2/TRPV4/ASIC modulators, or hormonal regulation.

## 4. Materials and Methods

### 4.1. Animals

C57BL/6J mice (WT, Jackson Laboratory) and Nav1.8-ChR2/eYFP mice were used in experiments. Nav1.8-ChR2/eYFP mice were generated by crossing Scn10a-Cre (Nav1.8-Cre) and Ai32 (RCL-ChR2(H134R)/eYFP) transgenic mice. Scn10a-Cre mice were gifts from Dr. John Wood at University College London and transferred to us from Dr. Stephen Waxman’s lab at Yale University. Ai32 mice were purchased from Jackson Labs. We performed a crossbreeding of Nav1.8-Cre mice with Ai32 (RCL-ChR2(H134R)/eYFP) mice to establish the Nav1.8-Cre+; ChR2/eYFP loxP/+ mouse line, referred to as Nav1.8-ChR2/eYFP hereafter. These mice express eYFP in their Nav1.8-positive (Nav1.8^+^) TG neurons, which are mainly nociceptors [30]. The mice used in the present study were aged 13–21 weeks, and both male and female mice were included. All animals were housed in a temperature-controlled room at 23 °C and maintained on a 12 h light/dark cycle. All animal care and experimental procedures were conducted following the guidelines established by the National Institutes of Health (NIH) for the care and use of experimental animals. Approval for the experimental protocols employed in this study was granted by the Institutional Animal Care and Use Committee (IACUC) at the University of Alabama at Birmingham.

### 4.2. Retrograde Labeling of Maxillary Sinus Neurons with Fluorescent Dye DiD

The fluorescent dye DiD (1,1′-dioctadecyl-3,3,3′,3′-tetramethylindocarbocyanine perchlorate; Invitrogen) was prepared as a 1 mM stock solution in 100% ethanol and stored at −20 °C, protected from light. Just before use, the stock was diluted in sterile 0.9% saline to a working concentration of 0.5 mM, thoroughly vortexed, and kept on ice, shielded from light throughout the procedure. DiD injections were performed under aseptic conditions. In brief, mice were anesthetized with isoflurane (induction: 3–4%; maintenance: 1.5–2% in oxygen) and placed supine on a heating pad to maintain body temperature. Using a 10 µL Hamilton syringe fitted with a 34 G nanoneedle (JBP) and with the needle angled toward the lateral wall of the maxillary sinus, the DiD solution (3 µL) was injected into the maxillary sinus. The needle was held in place for approximately one minute to ensure proper delivery. A successful injection was confirmed by the absence of fluid leakage from the nasal cavity [45]. Following the injection, mice were monitored until they fully recovered from anesthesia.

### 4.3. Ex Vivo Trigeminal Ganglion Preparations and Patch-Clamp Recordings

Ex vivo TG preparations and patch-clamp recordings were made in a manner similar to our previous studies [46]. In brief, the C57BL/6J mice and Nav1.8-ChR2/eYFP mice were anesthetized and decapitated, and trigeminal ganglia (TG) were bilaterally dissected out with attached infraorbital (V2) nerve bundles (5–7 mm) and submerged in ice cold Krebs solution in a 35 mm Petri dish containing (in mM): 117 NaCl, 3.6 KCl, 1.2 Na_2_PO_4_, 2.5 CaCl_2_, 1.2 MgCl_2_, 25 NaHCO_3_, and 25 glucose. The Krebs solution was saturated with 95% O_2_ and 5% CO_2_, had a pH of 7.35, and an osmolarity of 324 mOsm. Connective tissues on the surface of the TG were removed with fine forceps, and the ex vivo TG preparation was then placed, with the ventral side facing up, in a recording chamber and affixed to the bottom of the chamber with a tissue anchor and submerged in the Krebs solution at the room temperature of 24 °C. The recording chamber was mounted on the stage of an Olympus BX51 microscope that was equipped with IR-DIC and fluorescent imaging systems. TGs were exposed to 0.06% dispase II (Roche, Indianapolis, IN, USA) and 0.06% collagenase (MilliporeSigma, Billerica, MA, USA) in the Krebs solution for 6 min at a room temperature of 24 °C. The enzymes were then washed with Krebs solution, and the TG preparation was continuously perfused at 2 mL/min with Krebs solution.

For patch-clamp recordings, electrodes were fabricated using a micropipette puller (Sutter Instruments, Novato, CA, USA). The electrode resistance ranged from 4 to 6 MΩ after filling the recording electrode internal solution. The recording electrode internal solution contained (in mM): 105 K-gluconate, 35 KCl, 0.5 CaCl_2_, 2.4 MgCl_2_, 5 EGTA, 10 HEPES, 5 Na_2_ATP, and 0.33 GTP-TRIS salt; the pH was adjusted to 7.35 with KOH. The junction potential was 12 mV, calculated from the ionic concentrations of the internal and bath solutions using pCLAMP 11 (Molecular Devices, San Jose, CA, USA). Under a fluorescent and DIC-IR microscope, DiD-labelled TG neurons innervating the maxillary sinus (maxillary sinus TG neurons) were visualized with a 640–680 nm fluorescent filter, and Nav1.8-ChR2/eYFP-positive (Na1.8^+^) TG neurons in the V2 regions of TGs were visualized with a GFP fluorescent filter (520–550 nm). The sinus TG neurons and small-sized Nav1.8^+^ TG neurons were selected for whole-cell patch-clamp recordings. After establishing whole-cell access, recordings were performed in current-clamp mode to classify TG neurons based on the action potential (AP) conduction velocity (CV) of their afferent fibers. APs were evoked at the peripheral end of afferent nerve bundles using a suction stimulation electrode. The suction stimulation electrode was fire-polished and had a tip diameter of ∼1 mm. The peripheral end of the afferent nerve was aspirated into the suction stimulation electrode under negative pressure. APs were evoked by monophasic square wave pulses generated by pClamp11 software (Version 11) and delivered via a stimulation isolator (ISO-Flex, A.M.P.I, Sequim WA, USA) to the peripheral ends of the afferent nerves within the suction stimulation electrode. The stimulation pulse duration was 50 µs. CV was calculated from AP latency and afferent nerve length. The latency of APs was measured from the time of stimulation, which was marked by a stimulation artifact, to the time when AP was initiated at the recorded TG neuron. The length of the afferent nerves between the site of electrical stimulation and the recording site was 5 to 7 mm. The afferents with CV < 1 m/s were considered C-afferents [30]. To determine the properties of membranes and action potentials of recorded TG neurons, patch-clamp recordings were performed under the whole-cell current-clamp configuration. Step currents were injected into TG neurons through recording electrodes. Step currents were applied from −10 pA to 300 pA in 10 pA increments, with a 500-ms step duration. APs evoked by current steps were used to determine AP parameters, including AP rheobase, AP amplitude, AP width, and AP thresholds. AP amplitude was measured from RMP to the peak of AP evoked at a minimum current step. AP afterhyperpolarization (AHP) was measured from RMP to the deepest hyperpolarization of the APs generated by electrical stimulation of the peripheral ends of the afferent nerves. To characterize ionic currents in trigeminal ganglion (TG) neurons, whole-cell patch-clamp recordings were performed in voltage-clamp mode, with the membrane potential held at −72 mV, corrected for a junction potential of −12 mV. Voltage steps ranged from −100 mV to +40 mV (command voltages from −88 mV to +52 mV) in 10 mV increments, with each step lasting 500 ms. Unless otherwise stated, all reported membrane voltages were corrected for a calculated junction potential of 12 mV. Signals were amplified with an Axopatch 200B amplifier, low-pass filtered at 2 kHz, and sampled at 20 kHz using pCLAMP 11 software (Molecular Devices). All recordings were conducted at 24 °C, with a holding potential of −72 mV (corresponding to a −60 mV command voltage).

### 4.4. Mechanical Stimulation

Sinus TG neurons were mechanically stimulated by displacing their membranes with a mechanical probe. The mechanical probe was fabricated from a glass pipette with the tip fire-polished, and the tip diameter was ∼approximately 2 μm. The mechanical probe was mounted on a pipette holder and controlled by a piezo actuator (Physik Instrumente, Auburn, MA, USA). In the experiment, whole-cell patch-clamp recording was first applied to a sinus TG neuron. Then, the mechanical probe was positioned at the site of stimulation on the surface of the recorded TG neuron. To initiate mechanical stimulation, the tip of the mechanical probe moved forward, at 30 degrees, to displace cell membranes with displacement steps each at a 1-μm increment for up to 10 μm. Each displacement step consisted of a step-up phase at 2 µm/ms, a 150 ms holding phase, and a step-down phase, the reversal of the step-up.

### 4.5. Dissociation of Individual Sinus TG Neurons and Single-Cell RT-PCR

Individual DiD-labeled TG neurons (sinus TG neurons) were collected from TGs of Nav1.8-ChR2/eYFP mice that were retrograde-labeled with dye DiD. In brief, 5 days following the microinjection of dye DiD into the maxillary sinus, Nav1.8-ChR2/eYFP mice were anesthetized and decapitated, and the TGs were bilaterally dissected out. Under a dissection microscope, V2 and V1 parts of TG (V2 TG and V1 TG) were identified, and a cut along the midline of TG was made to separate the V2 part from the neighboring V1 region of the TG. The V2 TG was cut into 5–6 pieces using scissors, then incubated with dispase II (6 mg/mL) and type I collagenase (6 mg/mL) in 300 µL Krebs solution at 35 °C for 10 min. The bath solution was the same as that used for cell perfusion in electrophysiological experiments. After a rinse, V2 TGs were triturated to dissociate the neurons in the bath solution, and the dissociated V2 TG neurons were allowed to settle down on the bottom of a glass Petri dish coated with poly-D-lysine at room temperature for 5 min. The cells were perfused with normal bath solution flowing at 1 mL/min in a 0.5-mL recording chamber placed on the stage of an Olympus IX70 microscope. The individual DiD-labeled TG neurons were visualized under the fluorescent microscope, and each of them was aspirated into a micropipette (tip diameter, ~15 µm). The micropipette tips containing single cells were broken off directly into sterile microcentrifuge tubes containing lysis buffer. For cell collection, 6 sinus TG neurons were harvested and pooled together in a testing tube for RT-PCR, which was counted as one observation. Total RNA was extracted from the collected single neurons using the PureLink™ RNA Mini Kit (Invitrogen, Waltham, MA, USA) according to the manufacturer’s protocol, on the same day as cell collection. cDNA was synthesized from total RNA using the iScript™ cDNA Synthesis Kit (Bio-Rad, Hercules, CA, USA) and stored at −20 °C until PCR amplification. Gene-specific primers were designed using the NCBI Primer-BLAST tool (https://www.ncbi.nlm.nih.gov/tools/primer-blast/ (accessed on 15 February 2026)) and verified for specificity using NCBI BLAST. All primers were synthesized by Eurofins. Primer sequences and expected product sizes were as follows:
*Gapdh*:Forward 5′-AACTTTGGCATTGTGGAAGG-3′,
Reverse 5′-ACACATTGGGGGTAGGAACA-3′ (223 bp)*Piezo2*:Forward 5′-ACGGTCCAGCTTCTCTTCAA-3′,
Reverse 5′-GTCAGCCAGAAACATCAGCA-3′ (271 bp)*Scn11a*:Forward 5′-GCTTTGGCTGGTCTTTTC-3′,
Reverse 5′-CCTCCTTTTCCTCCCTCAAC-3′ (270 bp)*Scn10a*:Forward 5′-ATGAGGGTAGTGGTGGATGCC-3′,
Reverse 5′-AAAATGGGTTGCTTCTGGTG-3′ (160 bp)

For Piezo2, the primer sequence was for the complete Mus musculus strain C57BL/6J piezo2 mRNA, complete cds, from NCBI (GenBank: HQ215521.1), and it covers all coding regions of the gene, including known splice variants.

Annealing temperatures and primer concentrations were optimized using gradient PCR. The PCR protocol included an initial denaturation at 94 °C for 5 min, followed by 40 cycles of denaturation at 94 °C for 15 s, annealing at 60–62 °C for 20 s, and extension at 72 °C for 30 s. PCR products were separated by gel electrophoresis and visualized using a UVP ChemStudio imaging system (Analytik Jena, Jena, Germany) with VisionWorks software (Version LS 7.0).

### 4.6. Data Analysis

All electrophysiological data from TG neurons were acquired using pClamp 11 software and analyzed offline with Clampfit 11 (Molecular Devices). Data from both sexes were pooled together as we did not observe a tendency for sex differences in the AP properties of maxillary sinus TG neurons. Neurons were included in the analysis only if stable recordings were maintained and the access resistance remained <15 MΩ throughout the recording. Schematic illustration (Figure 1A) was created with BioRender. Data are reported as mean ± SEM from *n* independent observations, for comparisons between two groups, unpaired or paired Student’s *t*-tests were used as appropriate. For multiple group comparisons, one-way ANOVA with Tukey’s post hoc test or two-way ANOVA with Šídák’s multiple comparisons test was performed using GraphPad Prism version 10.1.2 (GraphPad Software, San Diego, CA, USA). Differences were significant with * *p* < 0.05, ** *p* < 0.01, and *** *p* < 0.001, and not significant (ns) with *p* ≥ 0.05.

## Figures and Tables

**Table 1:** Comparison of Sensory Innervation and Ion Channels Across Nasal Tissues.

Category	Nasal Skin [41]	Nasal Mucosa [42,43]	Paranasal Sinus Mucosa (Findings of This Study)	Dura [44]
**Nerve types** [41]	**Aβ fibers**, abundant, heavily myelinated (fast touch/pressure); **Aδ fibers**, prominent, lightly myelinated (intermediate speed transmission of pain and temperature); **C fibers**, unmyelinated, slow, dull pain, itch, and thermal sensations.	**Aβ fibers**, sparse and minimal function; **Aδ fibers**, rapid pain/itch, present but less dominant; **C fibers** (polymodal), slow, burning pain, dominant.	**Aβ fibers**, not present; **Aδ fibers**, few; **C fibers**, dominant, provide sensation of pressure.	**Aβ fibers**, sparse and minimal function; **Aδ fibers**, present; **C fibers**, dominant, provide sensation of pain.
**Nociceptors**	A broad variety of encapsulated and unencapsulated sensory receptors (e.g., Meissner’s, Pacinian corpuscles for touch/pressure); Piezo2, touch sensors	**Free endings**, mechanosensitive ion channels, active nociceptors—present; silent nociceptors—dominant, solitary chemosensory cells detect chemical irritants, temperature, and mechanical stimuli	**Free endings**, mechanosensitive ion channels, active nociceptors—present; silent nociceptors—dominant.	**Free endings**, mechanosensitive ion channels, active nociceptors—present; silent nociceptors—dominant.
**Sodium ion channels**	Transmit sensations of touch, temperature, and pain. **Nav1.7**—tetrodotoxin-sensitive (TTX-S), initiate action potential; **Nav1.8**—tetrodotoxin-resistant (TTX-R), enhances propagation—major role in pain sensation; **Nav1.9**—(TTX-R), sets firing threshold.	**Nav1.7**—sets firing threshold, involved in inflammation and cough; **high Nav1.8**—regulates airway defense mechanisms and airway hyperreactivity; **lower Nav1.9**—regulating sensitivity, more prominent	**Nav1.7**, not present; **Nav1.8**, low expression; **Nav1.9**, high expression, promotes hypersensitivity & persistent pain	**Nav1.7**, high expression; **Nav1.8**, variable; **Nav1.9**, prominent.
**Mechanosensitive ion channels**	**Piezo2**, touch; **ASICs**, present; **P2X3**, present.	**Piezo2**, responds to tissue distortion, edema; **ASICs**, acidic sensing, prominent; **P2X3**, inflammation-related, on olfactory nerves, relevant, specialized	**Piezo2** (high expression); **TRPV4**, **ASICs**, or other mechanoreceptors). Respond to stretch, edema—senses pressure	**Piezo2** (high expression) **TRPV4** &**ASICs** (likely), responds to stretch/pulsation
**TRP (Transient Receptor Potential) cation channels**	**TRPV3**, **TRPV4**, prominent role in temperature sensation, high expression; **TRPV1**, promotes or suppresses inflammation; **TRPA1**, mediates pain, itch, and cold sensation; **TRPM8**, low expression.	**TRPV1**, responds to heat, capsaicin, irritants, inflammatory sensitization, more prominent; **TRPA1**, chemical irritants, oxidative stress, respond to allergens; **TRPM8**, cooling sensations (menthol sensitivity).		**TRPV1**, responds to inflammatory heat signaling; **TRPA1**, responds to oxidative/inflammatory stress, highly expressed; **TRPM8**, sparse.

## Data Availability

The original contributions presented in this study are included in the article. Further inquiries can be directed to the corresponding authors.

## References

[1] Lafci Fahrioglu S., VanKampen N., Andaloro C. (2025). Anatomy, Head and Neck, Sinus Function and Development. StatPearls.

[2] Whyte A., Boeddinghaus R. (2019). The maxillary sinus: Physiology, development and imaging anatomy. Dentomaxillofacial Radiol..

[3] De Corso E., Kar M., Cantone E., Lucidi D., Settimi S., Mele D., Salvati A., Muluk N.B., Paludetti G., Cingi C. (2018). Facial pain: Sinus or not?. Acta Otorhinolaryngol. Ital..

[4] Helliwell T. (2010). Inflammatory diseases of the nasal cavities and paranasal sinuses. Diagn. Histopathol..

[5] Dykewicz M.S., Hamilos D.L. (2010). Rhinitis and sinusitis. J. Allergy Clin. Immunol..

[6] Misirovs R., Mohamad S. (2020). Reverse Squeeze Maxillary Sinus Barotrauma. Ear Nose Throat J..

[7] Lee S., Lane A.P. (2011). Chronic rhinosinusitis as a multifactorial inflammatory disorder. Curr. Infect. Dis. Rep..

[8] Eross E., Dodick D., Eross M. (2007). The Sinus, Allergy and Migraine Study (SAMS). Headache.

[9] Kari E., DelGaudio J.M. (2008). Treatment of sinus headache as migraine: The diagnostic utility of triptans. Laryngoscope.

[10] Agius A.M., Jones N.S., Muscat R. (2014). Prospective three-year follow up of a cohort study of 240 patients with chronic facial pain. J. Laryngol. Otol..

[11] Lal D., Rounds A., Dodick D.W. (2015). Comprehensive management of patients presenting to the otolaryngologist for sinus pressure, pain, or headache. Laryngoscope.

[12] Ali A.H.A., Serhan O.O., Alsharif M.H.K., Elamin A.Y., Al-Ghamdi S., Aldossari K.K., Alrudian N., Alajmi M., Alhariqi B.A., Mokhatrish M. (2022). Incidental detection of paranasal sinuses abnormalities on CT imaging of the head in Saudi adult population. PLoS ONE.

[13] Liu Y., Peng X., Yuan X., Wang M., Geng C., Xing Z. Clinical Manifestations and Management Challenges in Symptomatic Sinonasal Disorders With Normal CT Scans.

[14] Cady R.K., Schreiber C.P. (2002). Sinus headache or migraine? Considerations in making a differential diagnosis. Neurology.

[15] Al-Hashel J.Y., Ahmed S.F., Alroughani R., Goadsby P.J. (2013). Migraine misdiagnosis as a sinusitis, a delay that can last for many years. J. Headache Pain.

[16] Schreiber C.P., Hutchinson S., Webster C.J., Ames M., Richardson M.S., Powers C. (2004). Prevalence of migraine in patients with a history of self-reported or physician-diagnosed “sinus” headache. Arch. Intern. Med..

[17] Kim J.R., Park T.J., Agapova M., Blumenfeld A., Smith J.H., Shah D., Devine B. (2025). Healthcare resource use and costs associated with the misdiagnosis of migraine. Headache.

[18] Kim H.K., Chung K.M., Xing J., Kim H.Y., Youn D.H. (2024). The Trigeminal Sensory System and Orofacial Pain. Int. J. Mol. Sci..

[19] Pena E., Berciano M.T., Fernandez R., Ojeda J.L., Lafarga M. (2001). Neuronal body size correlates with the number of nucleoli and Cajal bodies, and with the organization of the splicing machinery in rat trigeminal ganglion neurons. J. Comp. Neurol..

[20] Hameed S. (2019). Na(v)1.7 and Na(v)1.8: Role in the pathophysiology of pain. Mol. Pain.

[21] Luiz A.P., Kopach O., Santana-Varela S., Wood J.N. (2015). The role of Nav1.9 channel in the development of neuropathic orofacial pain associated with trigeminal neuralgia. Mol. Pain.

[22] Aust R., Falck B., Svanholm H. (1979). Studies of the gas exchange and pressure in the maxillary sinuses in normal and infected humans. Rhinology.

[23] Li Q., Wang Z., Wang C., Wang H.L. (2022). Characterizing the respiratory-induced mechanical stimulation at the maxillary sinus floor following sinus augmentation by computational fluid dynamics. Front. Bioeng. Biotechnol..

[24] Ikeda R., Cha M., Ling J., Jia Z., Coyle D., Gu J.G. (2014). Merkel cells transduce and encode tactile stimuli to drive Abeta-afferent impulses. Cell.

[25] Woo S.H., Ranade S., Weyer A.D., Dubin A.E., Baba Y., Qiu Z., Petrus M., Miyamoto T., Reddy K., Lumpkin E.A. (2014). Piezo2 is required for Merkel-cell mechanotransduction. Nature.

[26] Szczot M., Liljencrantz J., Ghitani N., Barik A., Lam R., Thompson J.H., Bharucha-Goebel D., Saade D., Necaise A., Donkervoort S. (2018). PIEZO2 mediates injury-induced tactile pain in mice and humans. Sci. Transl. Med..

[27] Ranade S.S., Woo S.H., Dubin A.E., Moshourab R.A., Wetzel C., Petrus M., Mathur J., Begay V., Coste B., Mainquist J. (2014). Piezo2 is the major transducer of mechanical forces for touch sensation in mice. Nature.

[28] Odem M.A., Bavencoffe A.G., Cassidy R.M., Lopez E.R., Tian J., Dessauer C.W., Walters E.T. (2018). Isolated nociceptors reveal multiple specializations for generating irregular ongoing activity associated with ongoing pain. Pain.

[29] Viatchenko-Karpinski V., Ling J., Gu J.G. (2018). Down-regulation of Kv4.3 channels and a-type K(+) currents in V2 trigeminal ganglion neurons of rats following oxaliplatin treatment. Mol. Pain.

[30] Yamada A., Yamada A.I., Ling J., Furue H., Luo W., Gu J.G. (2023). Properties of Nav1.8(ChR2)-positive and Nav1.8(ChR2)-negative afferent mechanoreceptors in the hindpaw glabrous skin of mice. Mol. Brain.

[31] Gee M.D., Lynn B., Basile S., Pierau F.K., Cotsell B. (1999). The relationship between axonal spike shape and functional modality in cutaneous C-fibres in the pig and rat. Neuroscience.

[32] Fang X., McMullan S., Lawson S.N., Djouhri L. (2005). Electrophysiological differences between nociceptive and non-nociceptive dorsal root ganglion neurones in the rat in vivo. J. Physiol..

[33] Koster P.A., Leipold E., Tigerholm J., Maxion A., Namer B., Stiehl T., Lampert A. (2025). Nociceptor sodium channels shape subthreshold phase, upstroke, and shoulder of action potentials. J. Gen. Physiol..

[34] Korner J., Lampert A. (2022). Functional subgroups of rat and human sensory neurons: A systematic review of electrophysiological properties. Pflug. Arch..

[35] Ritter D.M., Ho C., O’Leary M.E., Covarrubias M. (2012). Modulation of Kv3.4 channel N-type inactivation by protein kinase C shapes the action potential in dorsal root ganglion neurons. J. Physiol..

[36] Ho C., O’Leary M.E. (2011). Single-cell analysis of sodium channel expression in dorsal root ganglion neurons. Mol. Cell Neurosci..

[37] Bonnet C., Hao J., Osorio N., Donnet A., Penalba V., Ruel J., Delmas P. (2019). Maladaptive activation of Nav1.9 channels by nitric oxide causes triptan-induced medication overuse headache. Nat. Commun..

[38] Carvalho T., Mello J.F., Caldini E., Salgado D.C., Carvalho N.M.G., Damaceno-Rodrigues N.R., Voegels R.L. (2023). Perivascular Innervation in the Nasal Mucosa and Clinical Findings in Patients with Allergic Rhinitis and Idiopathic Rhinitis. Int. Arch. Otorhinolaryngol..

[39] Maggi C.A. (1995). Tachykinins and calcitonin gene-related peptide (CGRP) as co-transmitters released from peripheral endings of sensory nerves. Prog. Neurobiol..

[40] Murthy S.E., Loud M.C., Daou I., Marshall K.L., Schwaller F., Kuhnemund J., Francisco A.G., Keenan W.T., Dubin A.E., Lewin G.R. (2018). The mechanosensitive ion channel Piezo2 mediates sensitivity to mechanical pain in mice. Sci. Transl. Med..

[41] Yam M.F., Loh Y.C., Tan C.S., Khadijah Adam S., Abdul Manan N., Basir R. (2018). General Pathways of Pain Sensation and the Major Neurotransmitters Involved in Pain Regulation. Int. J. Mol. Sci..

[42] Cong J., Lv H., Xu Y. (2024). The role of nociceptive neurons in allergic rhinitis. Front. Immunol..

[43] Shusterman D. (2023). Trigeminal Function in Sino-Nasal Health and Disease. Biomedicines.

[44] Huang D., Li S., Dhaka A., Story G.M., Cao Y.Q. (2012). Expression of the transient receptor potential channels TRPV1, TRPA1 and TRPM8 in mouse trigeminal primary afferent neurons innervating the dura. Mol. Pain.

[45] Gelbard A., Kupferman M.E., Jasser S.A., Chen W., El-Naggar A.K., Myers J.N., Hanna E.Y. (2008). An orthotopic murine model of sinonasal malignancy. Clin. Cancer Res..

[46] Okutsu Y., Yamada A., Tonomura S., Vaden R.J., Gu J.G. (2021). Electrophysiological properties of maxillary trigeminal Abeta-afferent neurons of rats. Mol. Pain.

